# Induction of twin pregnancy and the risk of caesarean delivery: a cohort study

**DOI:** 10.1186/s12884-015-0566-4

**Published:** 2015-06-16

**Authors:** Maria Jonsson

**Affiliations:** Department of Women’s and Children’s Health, Uppsala University, SE-751 85 Uppsala, Sweden

**Keywords:** Bishop score, Caesarean section, Induction, Induction method, Labour, Twin delivery, Twin pregnancy

## Abstract

**Background:**

Complications are common in twin pregnancies and induction of labour is often indicated. Most methods for induction are used but data on risks related to induction methods are sparse. The aim of this study was to investigate the association between induction of labour and caesarean delivery in twin pregnancies, and to assess the influence of induction method.

**Methods:**

Cohort study of twin pregnancies ≥ 34 weeks, planned for vaginal delivery, from two University Hospitals in Sweden. Data were collected from medical records during the periods 1994 (Örebro) and 2004 (Uppsala) to 2013. During the study period there were 78,180 live born births and 1,282 were twin births. Women with previous caesarean section were excluded. Induction methods were categorized into amniotomy, oxytocin and cervical ripening (intra cervical Foley catheter or prostaglandin). Adjusted odds ratios (AOR) with 95 % confidence interval (CI) for caesarean section were calculated by logistic regression and were adjusted for parity, maternal age, gestational length, complications to the pregnancy, infant birth weight and year of birth. Spontaneous labour onsets were used as the reference group. The main outcome measure was caesarean section.

**Results:**

In 462 twin pregnancies, 220 (48 %) had induction of labour and 242 (52 %) a spontaneous labour onset. Amniotomy was performed in 149 (68 %) of these inductions, oxytocin was administered in 11 (5 %) and cervical ripening was used in 60 (27 %). The rate of caesarean sections was 21 % in induced and 12 % in spontaneous labours (*p* 0.01). The absolute risk of caesarean section following induction was: 15 % with amniotomy; 36 % with oxytocin and 37 % with Foley/prostaglandin. Induction of labour increased the risk of caesarean section by 90 % compared with spontaneous labour onset (AOR 1.9, 95 % CI 1.1-3.5) and, when cervical ripening was used, the risk increased more than two fold (AOR 2.5, 95 % CI 1.2-5.3).

**Conclusion:**

Induction of labour in twin pregnancies increases the risk of caesarean section compared with spontaneous labour onset, especially if Foley catheter or prostaglandins are required. However, approximately 80 % of induced labours are delivered vaginally.

## Background

Numerous methods for induction of labour have been used in twin pregnancies, but data on safety and efficacy are limited. Published studies of this subject are few in number and small in size and, to date, results and experiences from labour induction of singletons are extrapolated to twins. Standards established for single gestations may not be applicable to twin gestations, since the circumstances for labour and delivery differ. For example, the progress of active phase labour is slower with twins for both nulli-and multiparous women [1] and slower labours in twins may be the result of uterine inertia due to an overdistended uterus and/or malpresentations [2].

Compared with singletons, twin pregnancies are associated with increased risks of complications such as gestational diabetes [3], gestational hypertension [4, 5], preeclampsia [6, 7], and intra hepatic cholestasis [8]. There is also an increased risk of stillbirth with advancing gestational age in otherwise uncomplicated twin pregnancies, which is why elective birth is recommended at 37–38 weeks gestation [9]. Thus, complications to twin pregnancies are frequently seen and labour induction is often indicated.

The potential advantages of induction of labour have to be balanced against any associated adverse consequences, such as an increased risk of caesarean delivery. The risk of caesarean section with induction of labour is, in part, a function of cervical ripeness, in that a higher rate of caesareans has been observed in women with lower Bishop scores compared to women with higher Bishop scores [10, 11]. With an unripe cervix, ways of promoting cervical ripening are needed and, in twin pregnancies, Foley catheters and various forms of prostaglandins are used [2].

For twin births, there is a limited amount of data about the risk of caesarean section by induction method. In previous observational studies, that included at the most 134 twin pregnancies, oxytocin, vaginal misoprostol and Foley catheters were used for induction and no difference in caesarean section rate was found when compared to singletons [12], oxytocin only [13] or expectant management [14]. It appears that previous studies dealing with induction of twin pregnancies are underpowered to address outcomes such as caesarean section. There are no studies with a sample size large enough to separate inductions that require cervical ripening agents from those allowing amniotomy, when estimating risk of caesarean section.

The aim of this study was to investigate the association between induction of labour and caesarean section through comparison of induction with spontaneous labour onset in twin pregnancies. A further aim was to analyse to what extent the association was influenced by method of induction.

## Methods

**Fig. 1:**
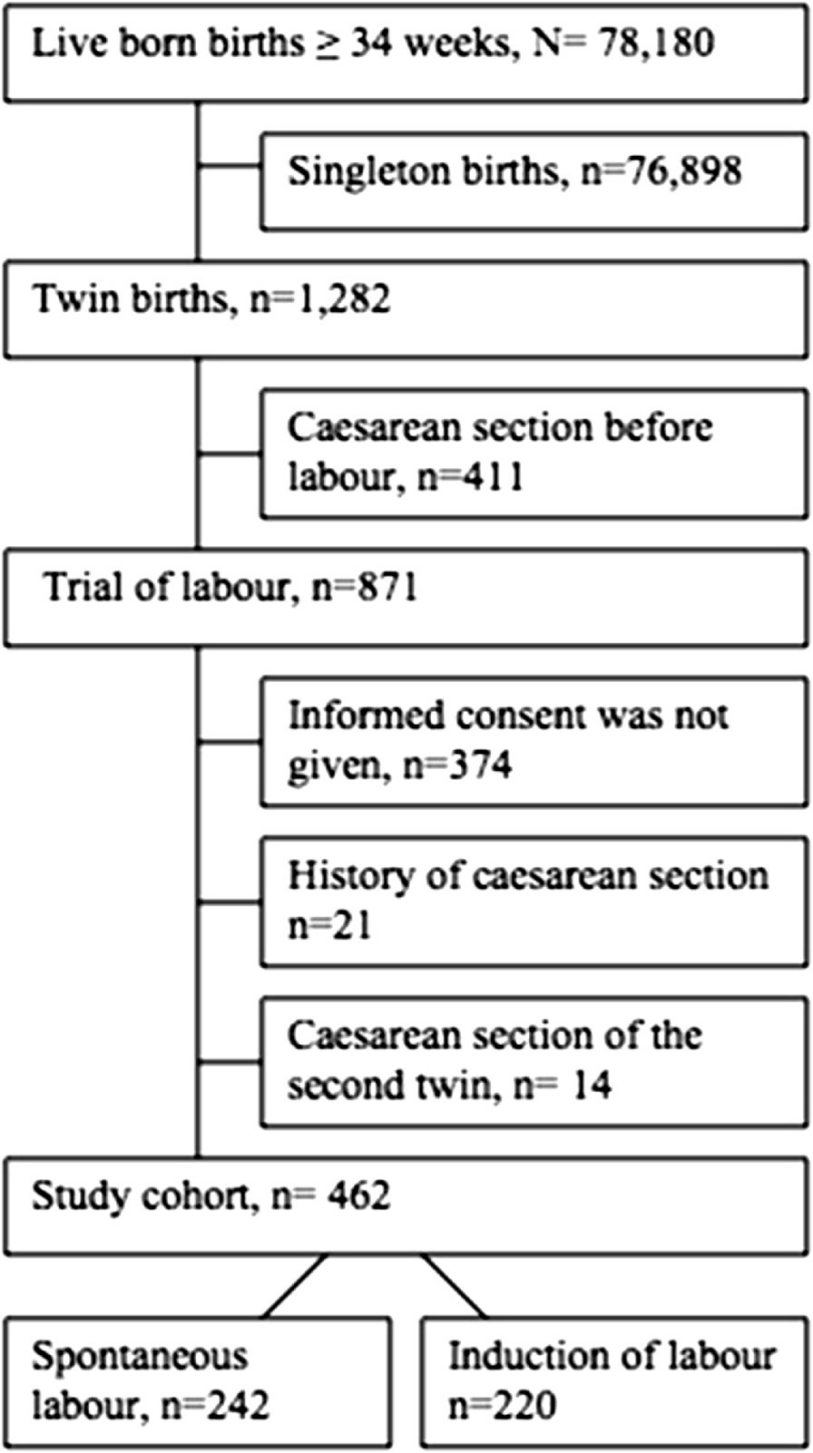
Flow chart of the study population

This is a cohort study on twin pregnancies at gestational age ≥34 weeks from two university hospitals in Sweden. All twin pregnancies were retrieved from a local database of both departments for the years 2004–2013 in Uppsala and 1994-2013 in Örebro. During the study period there were 78,180 live born births and the study cohort included 1,282 twin births (Uppsala: *n* 577; Örebro: *n* 705). Fig. 1 illustrates the flowchart of the study population. The regional Ethics Committee at Uppsala University approved the study and informed written consent was obtained from participants.

During the study period there was an increase in the total caesarean section rate at both units (12 % to 17 %) and the overall induction rate increased from 8 % in 1994 to 15 % in 2013. The caesarean section rate in twin deliveries increased by 10 %, and was 55 % in 2013.

Three hundred and seventy four women did not give informed consent, leaving 57 % of eligible cases to be included in the study. Twin gestations with caesarean section performed before labour, and women with a history of previous caesarean section were excluded. Births with a caesarean section of the second twin after vaginal delivery of the first twin were also excluded. The reason for exclusion was that caesarean delivery of the second twin after vaginal birth of the first twin is often performed due to malpresentation or threatened asphyxia, situations, that can occur regardless of how labour proceeded until delivery of the first twin. After exclusions, there were 462 women with twin pregnancies that formed the study cohort (Fig. 1).

Demographic information and characteristics of pregnancy, labour, delivery and neonatal data were retrieved by manual review of the medical records. Parity was categorized into nulliparous and parous, and maternal age into < 35 and 35 years or older. Gestational age was based on the estimated delivery date by ultrasound examinations routinely performed in the second trimester (around the 17th week of gestation), when chorionicity was also decided. A second trimester scan is routine in Sweden since the mid-90s and approximately 95 % of pregnant women are scanned. A first trimester scan is not routine in Sweden why chorionicity diagnosis is less accurate in our population. Embryo transfer determined gestational age when pregnancy was achieved by *in vitro* fertilization. Gestational length was categorized into < 36, 36–38 and >38 weeks. Complications during the pregnancy included gestational hypertension, preeclampsia, gestational diabetes, intrahepatic cholestasis, anaemia, intrauterine growth retardation and oligohydramniosis.

At the departments, cervical ripening, with the use of intracervical Foley catheter or vaginal prostaglandin E_2_ (dinoprostone), is generally applied if the Bishop score is less than six. From 2013, oral prostaglandin E_1_ (misoprostol) was used. Once Bishop score of six or more is reached, amniotomy is performed and oxytocin infusion started in most women that required cervical ripening. Amniotomy is performed with a Bishop score of six or more and usually oxytocin is administered within one to two hours. Oxytocin is used for induction of women with premature rupture of the membranes. The units used the same standard protocol for oxytocin. Inductions of labour were categorized into amniotomy, oxytocin or use of cervical ripening agent (Foley catheter or Prostaglandin).

Failed induction was considered the indication when a caesarean section was performed in the latency phase. Duration of labour was calculated from partograms, from onset of the active phase of labour (four cm cervical dilation) to delivery. Long labours were defined as lasting more than 12 hours. A progress of less than one cm cervical dilation per hour during at least four hours in active first stage labour or, a second stage lasting more than three hours, defined abnormal labour.

Birth weight of the neonates was categorized based on the 25^th^ and 75^th^ percentiles of the study population. In Sweden, small for gestational age (SGA) is defined as a birth weight less than-2 standard deviations (SD) from the mean for gestational age and gender. Umbilical artery sampling and blood gas analysis is routine at both departments. The main outcome was caesarean delivery.

Statistical analysis was performed with the SPSS statistical package 20.0 (SPSS Inc., Chicago, IL, USA). Differences between categorical variables were analysed using chi-square test. Differences between continuous variables were tested with the independent sample *t* test or a Mann–Whitney *U* test, where appropriate. Risk of caesarean delivery was estimated for births exposed for induction of labour, using spontaneous onset of labour as reference. Odds ratios (OR) are presented with 95 % confidence intervals (CI). The model was adjusted for parity, maternal age, complications to the pregnancy, gestational length in weeks, infant weight in percentiles (as described above), and year of birth categorized into before 2004 and 2004 or later.

## Results

Of 462 included twin births, 242 (52 %) had a spontaneous onset of labour and 220 (48 %) were induced. Indications for inductions were: gestational length (>38 weeks) 61 (28 %); preeclampsia 49 (22 %); elective 42 (19 %); intrauterine growth retardation 33 (15 %); intrahepatic cholestasis 11 (5 %); premature rupture of membranes 12 (5.5 %); gestational hypertension 6 (3 %) and others 6 (3 %). Elective inductions comprised women with a wish to end their pregnancy because of physical discomfort and tiredness.

The Bishop score in induced labours was less than six in 30 % of women with a median (interquartile range) of 6 (5–7). Inductions were started by amniotomy in 149 (68 %), oxytocin infusion in 11 (5 %) and Foley catheter/prostaglandin in 60 (27 %).

Maternal characteristics and data on labour and delivery are presented in Table 1. Compared with spontaneous onsets, women with induced labours had more advanced gestational age and more often complications to the pregnancy. Oxytocin was used more often and for a longer time in induced labours but there was no difference in maximum doses. Duration of labour or occurrence of protracted or long labours did not differ. The rate of caesarean sections was higher in induced compared with spontaneous labours, 21 % *vs.* 12 % respectively (p 0.01). There was no difference in the rate of vacuum deliveries. Forceps deliveries were not found in this cohort.

**Table 1:** Summary of maternal characteristics and data on labour and delivery according to labour onset

Characteristics	Induction of labour N = 220	Spontaneous labour N = 242	p value
Primipara	95 (43)	102 (42)	0.82
Age (years)	31 ± 4.5	31 ± 4.4	0.91
≥35	56 (26)	63 (26)	
<35	163 (74)	179 (74)	
Body mass index (kg/m^2^)	25 ± 5	26 ± 22	0.29
Height (cm)	166 ± 17	166 ± 12	085
Intecurrent disease	32 (15)	29 (11)	0.47
Smoking during pregnancy	15 (7)	27 (12)	0.10
Assisted fertilization	28 (13)	49 (21)	0.03
Dichoriotic diamniotic twins	160 (76)	181 (77)	0.62
Gestational length (weeks)	38 ± 2	36 ± 2	<0.001
<36	15 (7)	76 (31)	
36-38	143 (65)	136 (56)	
>38	62 (28)	30 (12)	
Complication to the pregnancy^1^	93 (42)	53 (22)	<0.001
Premature rupture of membranes	9 (4)	14 (6)	0.39
Epidural	87 (41)	89 (38)	0.55
Oxytocin	186 (84)	176 (73)	0.002
Oxytocin, minutes	300 (120–550)	100 (42–237)	<0.001
Protracted labour	44 (20)	51 (21)	0.88
Duration of labour, minutes	244 (132–404)	225 (121–426)	0.63
First stage	195 (95–330)	162 (90–330)	0.32
Second stage^2^	30 (14–82)	30 (16–87)	0.30
Bearing down	16 (8–30)	15 (7–29)	0.50
Long labour (>12 hours)	9 (5)	12 (6)	0.66
Vacuum delivery	37 (17)	36 (15)	0.57
Caesarean section	47 (21)	30 (12)	0.01
Breech 1^st^ twin	5 (2)	14 (6)	0.13
Breech 2^nd^ twin	65 (29)	71 (29)	0.97

Data are presented as n (%), mean (standard deviation) and median (interquartile range)

^1^Preeclampsia, hypertension, diabetes, intrahepatic cholestasis, intrauterine growth retardation, anaemia, and oligohydramniosis

^2^Includes passive and active (bearing down) second stage

Mean birth weights of the first and second twins were higher in induced pregnancies, but second twins were more often small for gestational age (Table 2). There were no differences in sex, Apgar score less than seven at five minutes or umbilical artery pH values at birth.

**Table 2:** Neonatal outcome

Variable	Induction of labour N = 220	Spontaneous labour N = 242	p value
First twin			
Female sex	101 (46)	129 (53)	0.11
Birth weight, g	2888 ± 431	2683 ± 476	<0.001
Small for gestational age^1^	22 (10)	28 (12)	0.57
Apgar score < 7 at 5 minutes	3 (1.4)	3 (1.2)	0.90
Umbilical artery pH	7.27 ± 0.4	7.30 ± 0.1	0.29
Second twin			
Female sex	115 (51)	112 (46)	0.36
Birth weight, g	2844 ± 470	2689 ± 462	<0.001
Small for gestational age^1^	33 (15)	18 (7)	0.01
Apgar score < 7 at five minutes	5 (2.3)	6 (2.5)	0.90
Umbilical artery pH	7.21 ± 0.6	7.24 ± 0.1	0.36

Data are presented as n (%) and mean (standard deviation)

^1^Defined as more or less than ± 2 standard deviations (SD) of mean birth weight

In labours induced by amniotomy or by Foley/prostaglandins, the latency phase had a median duration of, respectively, 150 (40–225) minutes, and 690 (442–1618) minutes, *p* < 0.001 (data not shown in Table). A Bishop score < 6 resulted in a caesarean section in 25 (38 %) women compared with 19 (12 %) when the Bishop score ≥ 6 (p < 0.001). Bishop score was missing in three cases (data not shown in table). Compared with spontaneous labour, the risk of caesarean section in induced labours was estimated to an adjusted odds ratio (AOR) of 1.9 with 95 % confidence interval (CI) 1.1 to 3.5 (Table 3). Nulliparity was the strongest risk factor for caesarean section. Gestational length, complications to the pregnancy and infant birth weight were not associated with an increased risk for caesarean section.

**Table 3:** Unadjusted and adjusted odds ratios (OR) for caesarean section by onset of labour and maternal and infant characteristics

Characteristics	Total number	CAESAREAN SECTION		
		N	Unadjusted OR (95 % CI)	Adjusted^1^ OR (95 % CI)
Labour onset				
Spontaneous	242	30	reference	reference
Induction	220	47	1.9 (1.1-3.2)	1.9 (1.1-3.5)
Parity				
≥1	265	13	reference	reference
0	197	64	9.3 (4.9-17.5)	14 (6.8-29)
Age (years)				
<35	343	56	reference	reference
≥35	119	21	1.1 (0.6-1.9)	1.9 (1.1-3.7)
Gestational length (weeks)				
<36	91	15	1.2 (0.6-2.4)	1.2 (0.5-3.1)
36-38	279	24	reference	reference
>38	92	38	2.2 (1.2-3.9)	1.8 (0.9-3.9)
Complication to the pregnancy				
No	316	46	reference	reference
Yes	146	28	1.6 (0.9-2.6)	1.1 (0.6-1.8)
Birth weight 1st twin (g)				
<2474	115	21	1.3 (0.7-2.3)	1.1 (0.5-2.3)
2474-3050	235	35	reference	reference
>3050	112	21	1.3 (0.7–2.4)	1.7 (0.8-3.6)
Birth weight 2nd twin (g)				
<2449	115	21	1.2 (0.7-2.3)	1.1 (0.5-2.6)
2449-3075	233	34	reference	reference
>3075	114	23	1.5 (0.8-2.6)	1.6 (0.8-3.4)

^1^Adjusted for maternal age, gestational length, parity, complications to the pregnancy, weight of first twin (percentiles) and year of birth

The indications for caesarean delivery in spontaneous labours were: suspected fetal asphyxia 23 (77 %); protracted labour 5 (16 %); one placental abruption and one transverse lie. In induced labours the indications were: suspected fetal asphyxia 26 (55 %); protracted labour 5 (11 %); and failed induction 16 (34 %).

Table 4 depicts the AOR of caesarean section when induced labour onsets were stratified into induction with amniotomy, oxytocin and cervical ripening. Odds ratios were adjusted for maternal age, gestational length, parity, complications to the pregnancy, weight of first twin and second twin and year of birth. Compared with spontaneous labour onsets, labours induced with cervical ripening were associated with a 2.5 times increased risk of caesarean section.

**Table 4:** Unadjusted and adjusted odds ratios (OR) for caesarean section by induction method

Onset of labour	Total number	CAESAREAN SECTION			
		N	Rate	Unadjusted OR (95 % CI)	Adjusted OR* (95 % CI)
Spontaneous	242	30	12	reference	reference
Amniotomy	149	21	15	1.2 (0.6-2.1)	1.4 (0.7-2.8)
Oxytocin	11	4	36	3.0 (0.7-12)	2.7 (0.5-14)
Foley/prostaglandin	60	22	37	4.1 (2.2-7.8)	2.5 (1.2-5.3)

*Adjusted for maternal age, gestational length, parity, complication to the pregnancy, weight of first and second twin (percentiles) and year of birth

## Discussion

This study suggests that induction of twin pregnancies is associated with a two-fold increase in risk of caesarean section compared with spontaneous labour onset, and that the increase in risk is associated to the need for cervical ripening. Despite the increased risk, the majority had a vaginal delivery. In women where amniotomy was possible, induction of labour had no increased risk of caesarean delivery. Almost half of pregnancies were induced in this study; the majority had a medical indication and were therefore necessary despite the increased risk of caesarean section. However, approximately 20 % had an elective induction and, women who want induction when there is no medical reason should be counselled about the benefit of postponing induction until amniotomy is possible.

The study is limited by the retrospective design and thus prone to potential confounding. By reviewing all records and by detailed collection of data on maternal, labour and delivery characteristics, an attempt was made to control for confounders, but some may still have been unidentified. Given that a substantial number of women declined participation, the possibility of selection bias must be recognized. To control for changes in management over time, year of delivery was controlled for in the regression analysis. Examples of changes during the study period include: the caesarean section rate before labour has increased; and twin deliveries are planned from 38 instead of 40 weeks’ gestational age. Generalization of the results from the predominantly Scandinavian population to other nationalities may be limited.

This is the first study on twin pregnancies that reports on the risk for caesarean section by induction method. However, women with spontaneous labour onset were used as a control group when the actual alternative is expectant management [15]. Retrospective cohort studies on elective induction (i.e. without medical induction) of labour in a specific week, compared to expectant management in single gestations at term, have not found a difference in risk for caesarean section from 37 weeks gestational age [16–20], this is consistent with results from randomized controlled trials [21, 22]. When induction of twin pregnancies has been compared with expectant management, no difference in caesarean section rates have been found, albeit the sample sizes were too small to address the outcome in these studies [14, 23]. Results from singleton pregnancies may not be directly translated to twin pregnancies and, to date, studies large enough to compare expectant management with induction of labour in twin gestations are lacking.

Data on induction of twin pregnancies are limited in the literature and information on the risk of caesarean section related to induction methods is sparse. In a retrospective observational study of induced twin pregnancies, a non-significant higher caesarean delivery rate with vaginally administered misoprostol (n = 57), compared with oxytocin (n = 77), was found [13]. The authors of that study speculated that the higher caesarean section rate was due to the higher rate of nulliparity and unripe cervical status in the misoprostol group. The present study confirms that nulliparity, as well as unripe cervical status, are independent risk factors for caesarean section in induced twin deliveries. In one study of twin gestations, in which outcomes of induced labours (n = 36) were compared with expectant management (n = 45), oxytocin was used in half of induced labours and Foley catheter or vaginal application of Prostaglandin E_2_ in the other half [14]. There was no difference in caesarean section rates; however, in that study, inductions were not stratified by method used. Another small study of 36 twin pregnancies, randomized to expectant management or induction with oral prostaglandin E_2_ at 37 gestational weeks, found a non–significant lower rate of caesarean deliveries in the induction group [23]. There were significantly more women with pre labour rupture of membranes in the expectant management group, indicating that the randomization did not result in comparable groups in that study, and maternal infection was the most common indication for caesarean section.

Failed induction, with a caesarean section performed in the latency phase, was a common indication for caesarean section (31 %) in the present study. Studies on the latency phase in twin gestations are sparse and contradictory. One study reports the latent phase to be shorter in twin gestations compared with singletons [24], unlike the finding in a recent cohort study, where twin gestations had significantly less cervical dilation at admission [1]. In singleton gestations, there is data to suggest that latent phase duration is longer in induced compared with spontaneous labour, whereas duration of the active phase is similar [25]. An arrest diagnosis before 6 cm dilation in induced labour is thought better avoided [25] and, by allowing longer duration of the latent phase, unnecessary caesarean deliveries for failed induction can probably be avoided [26].

Twin gestations have slower labour progress compared to singletons, regardless of parity, and require 1–3 hours more to complete the first stage of labour compared with singletons, according to a study by Leftwich and colleagues [1]. The authors of the study propose that patience in providers and patients, concerning the time needed to come through the active phase of twin labours, may reduce caesarean deliveries for failure to progress. Sub analysis of induced labours was not done in that study, although approximately 40 % of labours were induced in both groups and, information on induction method was not described [1]. The results of the present study indicate that when induced labour reaches the active phase, the progress is comparable to those with a spontaneous labour onset. In support of this finding, no difference in mean labour duration was found in previous studies in which induction of twin gestations was compared to expectant management [14, 23].

Twin gestation is associated with an increased risk of morbidity to the neonate. Caesarean section before labour is believed to reduce this risk and is one reason for the increase in caesarean delivery rate seen worldwide [27, 28]. In a recently published randomized controlled trial including twins between 32 and 38 weeks, caesarean section before labour did not reduce the risk of adverse neonatal or maternal outcomes compared with planned vaginal delivery [29]. In contrast, evidence from observational studies suggest there may be some benefit of pre-labour caesarean section for neonates at 36 weeks gestational age and greater, although the absolute rate of adverse neonatal outcome was reported low [30–32]. For the woman, a scarred uterus is one important source of morbidity and increases the risk of caesarean section in subsequent deliveries [33, 34]. Given current evidence, a trial of labour in twin pregnancy is still considered a reasonable and safe option [29]. In case of induction, the present study might provide information that could be useful in the counselling of women with a twin gestation.

## Conclusion

Compared with spontaneous labour, there is no increase in risk of caesarean section with induction of labour when amniotomy is the method used. There is a possible increased risk of caesarean section associated with induction of labour when Foley catheter or prostaglandins are used, even though the majority will deliver vaginally. When the active phase of labour is reached, the progress is similar in spontaneous and induced labours.

## Abbreviations

AOR: Adjusted odds ratio
CI: Confidence interval
OR: Odds ratio
SD: Standard deviation
SGA: Small for gestational age
SPSS: Statistical package for social sciences
